# The Importance of Catheter Angiography in Computed Tomography Angiography-Negative Subarachnoid Hemorrhage

**DOI:** 10.7759/cureus.1254

**Published:** 2017-05-17

**Authors:** Ali S Haider, Caleb Gottlich, Anadjeet Khahera, Steven Vayalumkal, Umair Khan, Eliel N Arrey, Jacob Campbell, Richa Thakur, Sam Finn, Kennith F Layton

**Affiliations:** 1 Texas A&M College of Medicine; 2 School of Medicine, University of California; 3 School of Medicine, St. George's University; 4 School of Medicine, St. Georges University; 5 Houston Methodist Neurological Institute, Houston Methodist Hospital, Houston, TX; 6 HSS Management; 7 Department of Radiology, Baylor University Medical Center

**Keywords:** subarachnoid hemorrhage, angiography, catheter, cta, sah

## Abstract

Computed tomography angiography (CTA) has become an effective tool in the evaluation of patients with subarachnoid hemorrhage (SAH), but it still has limitations. Up to 15% of non-traumatic SAH cases are negative on CTA. The benefits of catheter angiography in the evaluation of certain cases of CTA-negative SAH have been previously demonstrated. Here, we present the case of a 48-year-old female who presented with headache and right-sided hemiparesis, who later became apneic and required intubation. A computed tomography (CT) scan of the head demonstrated a diffuse SAH. A CTA of the head and neck showed no vascular abnormality.* *Catheter angiography diagnosed a conical-shaped aneurysm at the left A1-A2 junction of the anterior communicating artery complex measuring 3.5 mm by 1 mm. The aneurysm was successfully treated with a craniotomy and microvascular clipping using a 4.7 mm curved Yasargil miniclip (Aesculap, Tuttlingen, Germany). This case illustrates the importance and benefit of catheter angiography in CTA-negative cases of SAH.

## Introduction

Subarachnoid hemorrhage (SAH) is commonly seen in the neurosurgical patient population. SAH not caused by a traumatic event is frequently due to an intracranial aneurysm rupture. Non-traumatic SAH is predicted to affect approximately 20,000-30,000 people per year in the United States; of those affected, there is up to a 45% mortality rate within one month [1]. Computed tomography angiography (CTA) has become a new standard of care in many facilities due to its less invasive nature and high negative predictive value (NPV); furthermore, CTA is even being suggested as the primary means of evaluation and diagnosis in patients presenting with SAH [2-5]. Although this is a useful tool and serves as an effective screening test, it has its limitations. Up to 15% of non-traumatic SAH cases are found to be negative on CTA; these presentations are referred to as idiopathic or angiogram-negative SAH [6]. Of these angiogram-negative cases, there is further stratification based on location of the hemorrhage and prognostic indication; the main subcategories are perimesencephalic SAH (PMH SAH) and aneurysmal type SAH. Of the two types, PMH SAH is generally viewed as having a better prognosis while non-PMH SAH has higher uncertainty due to the increased risk of re-bleeding and elevated levels of morbidity. Due to this increased risk, it is important to follow a negative CTA with catheter angiography in those cases that do not have a classic PMH SAH imaging pattern and clinical presentation. Multiple studies in the current literature corroborate the practice of foregoing follow-up catheter angiography for those patients that present with a PMH SAH; however, those with peripheral or diffuse bleeding would benefit from further evaluation with catheter angiography [1-3]. Here, we present the case of a 48-year-old female who presented with a sudden onset severe headache and was found to have non-PMH SAH on a head computed tomography (CT) scan with a negative CTA. However, subsequent catheter angiography revealed a small aneurysm of the anterior communicating artery that was occult on CTA, even in retrospect. This case serves as a meaningful example of the importance of catheter angiography in specific CTA-negative cases.

## Case presentation

**Figure 1:**
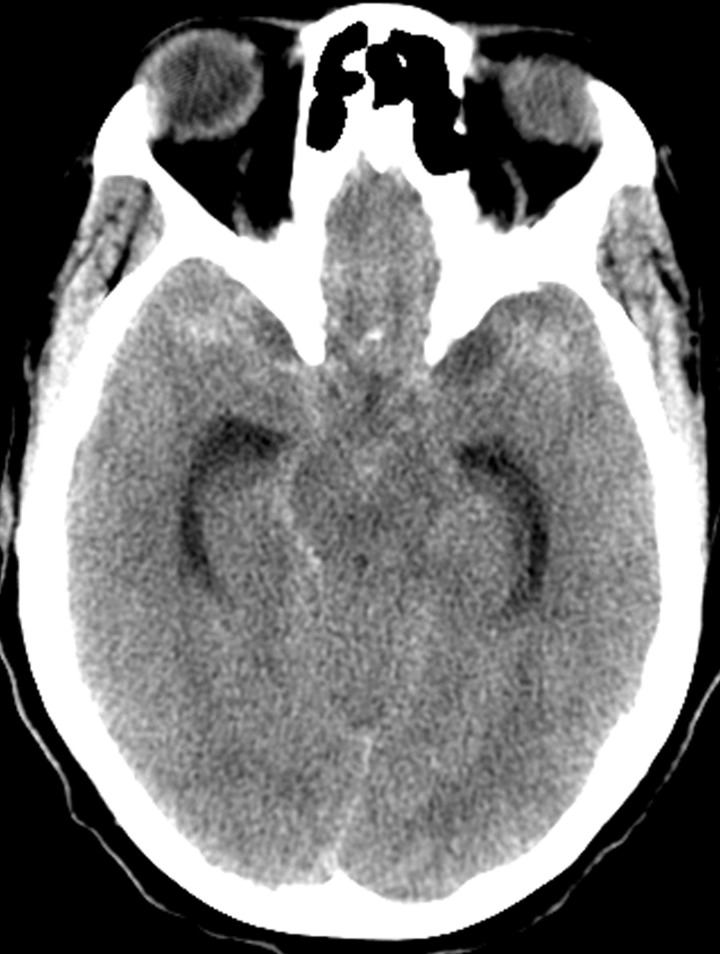
Non-contrast head computed tomography scan demonstrates diffuse bilateral subarachnoid hemorrhage. The volume and distribution of the blood is more than seen in a benign perimesencephalic hemorrhage.

A 48-year-old female with a past medical history of hypertension (HTN) was transferred to our cerebrovascular service from an outside institution for non-traumatic SAH. She had been in her usual state of health prior to the acute onset of severe headache and right sided hemiparesis. Her initial Glasgow Coma Scale score was between nine and ten at the outside emergency department. A head CT was obtained, which demonstrated a diffuse SAH (Figure 1).

**Figure 2:**
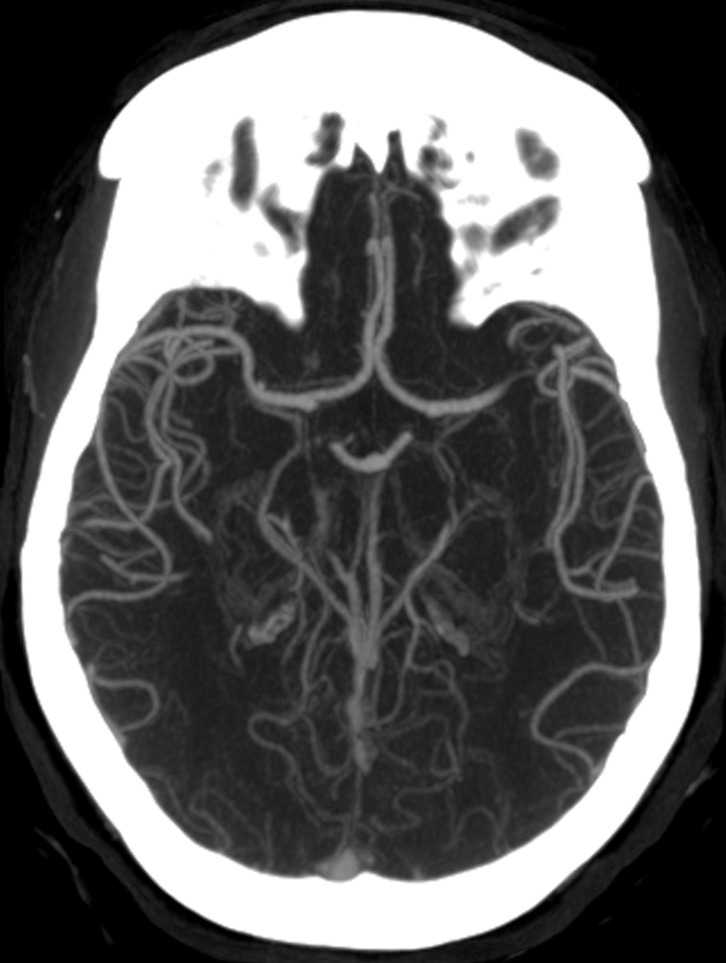
Axial computed tomography angiogram of the head performed with excellent technique through the region of the anterior communicating artery. Despite rigorous scrutiny by a neuroradiologist, no aneurysm was present.

During transport, the patient became apneic and required intubation by emergency medical personnel. She had no history of alcohol or drug use. On physical exam, she was able to follow verbal commands and had round and reactive pupils devoid of anisocoria, but she had no withdrawal to painful stimulus on the right foot. She had a clinical Hunt and Hess score of three and an imaging Fisher grade of three. Her labs showed an elevated serum glucose of 237, but the other values were within normal limits. A CTA was performed at our institution and was evaluated by a fellowship-trained diagnostic neuroradiologist whose practice is devoted solely to neuroradiology. Despite findings suspicious for a ruptured cerebral aneurysm, the neuroradiologist was not able to identify any vascular abnormality on the CTA (Figure 2).

**Figure 3:**
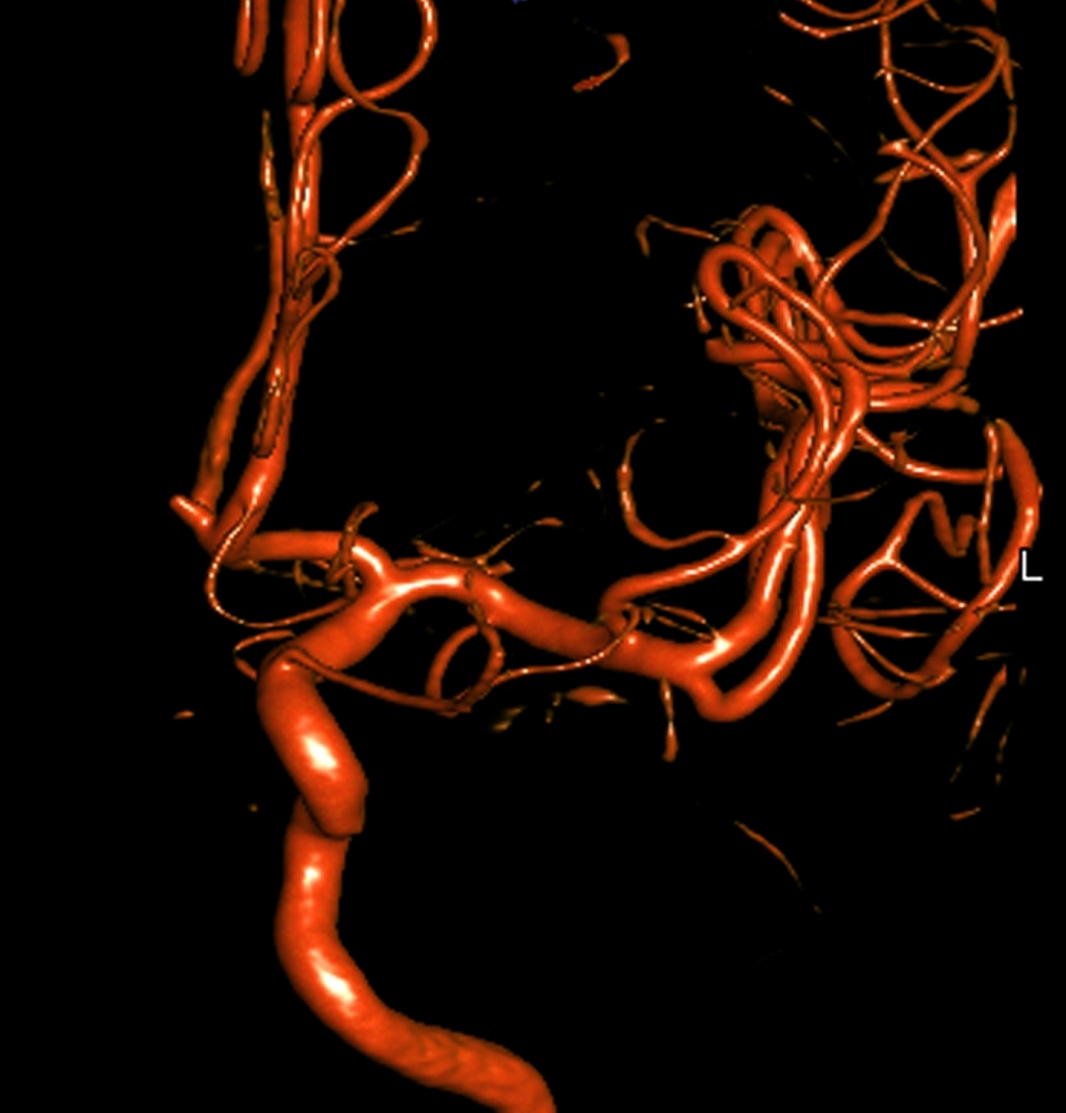
Left internal carotid artery injection from a digital subtraction catheter angiogram reveals a small aneurysm of the anterior communicating artery complex at the left A1-A2 junction. The patient's left side is designated by the white "L" in the image.

Therefore, the decision was made for the patient to undergo catheter angiography. Catheter angiography was performed by an experienced fellowship-trained interventional neuroradiologist, who diagnosed an aneurysm at the left A1-A2 junction of the anterior communicating artery complex. It was shown to be a conical-shaped aneurysm in the anterior communicating artery measuring 3.5 mm by 1 mm and was determined to be a poor candidate for coiling (Figure 3).

**Figure 4:**
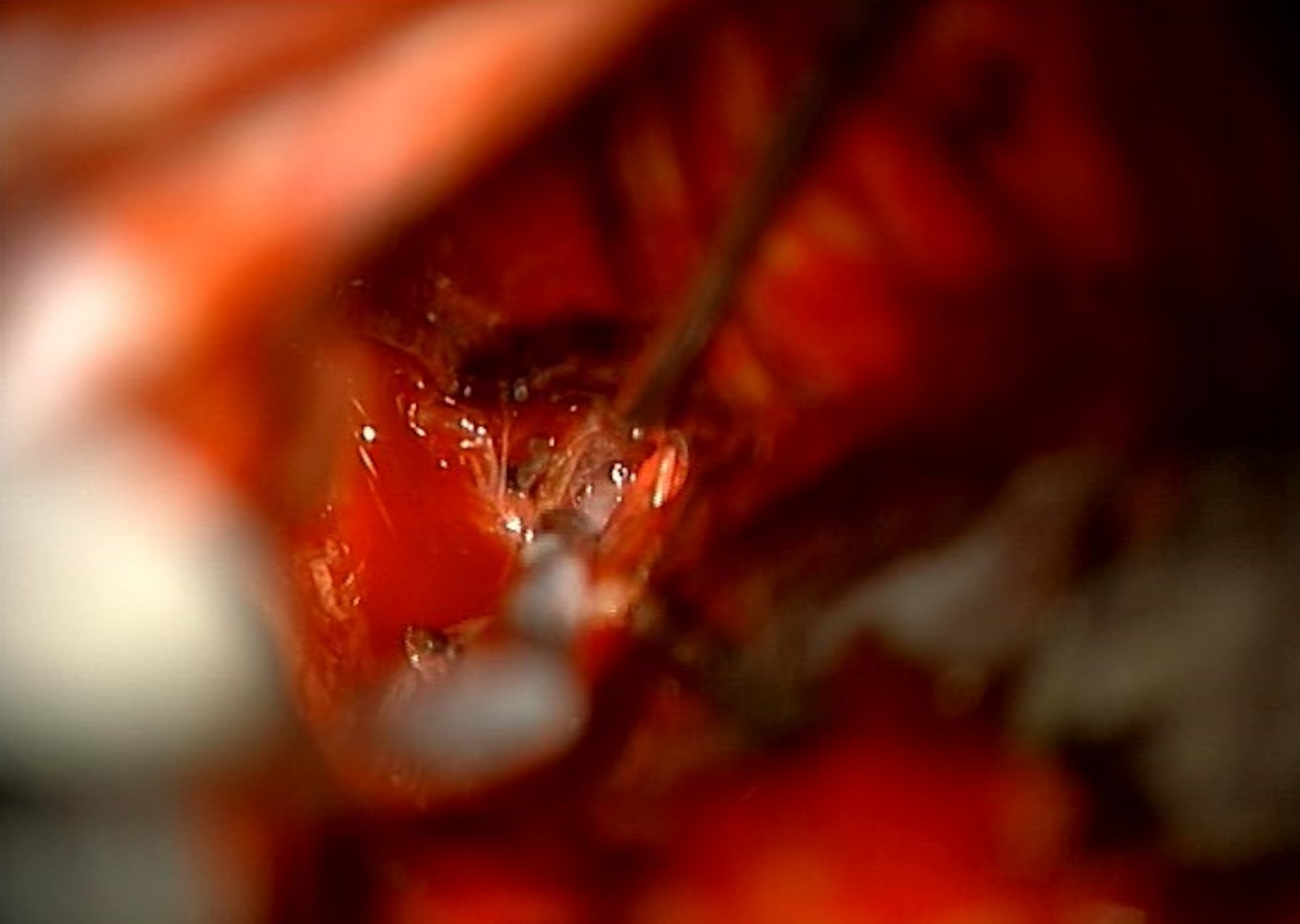
Intraoperative photograph during microsurgical clipping of the aneurysm. The decision to proceed with clipping was made by a consensus of the interventional neuroradiologist and the cerebrovascular neurosurgeon.

The aneurysm was successfully treated with a craniotomy and microvascular clipping using a 4.7 mm curved Yasargil miniclip (Aesculap, Tuttlingen, Germany) (Figure 4).

## Discussion

There is much to be gained from the proper use of CTA in non-traumatic SAH, assuming it is correctly performed and read by a fellowship-trained neuroradiologist. The protocol must be carried out meticulously, without patient motion and with good technical parameters to optimize the study. This includes the use of a modern multislice CT scanner and thin source images optimized for multiplanar reconstructions. Subsequent interpretation by a neuroradiologist with sufficient expertise in reading neurologic CTA studies at a high volume center is critical for test accuracy. Even then, opportunities for a false negative exam arise if the aneurysms are small or located next to bone or at a tortuous vessel loop, as was present in this case. There is a high risk of re-bleeding in unsecured ruptured aneurysms and its inherent morbidity and mortality far outweigh the minor risk imposed by catheter angiography performed by an experienced neurointerventionalist. There should be a heavy emphasis placed on the expertise of the radiologist reading the CTA in regard to the level of confidence that can be placed on a negative finding. One study showed a statistically significant discrepancy between residents, radiologists, and other consulting physicians reading CTAs in both the time taken to analyze and the quality of the analysis, providing a linear correlation to number of cases read and proficiency in ability [7]. This can be further extrapolated to apply to radiologists at an academic institution who have obtained additional post-residency training via a fellowship in neuroradiology and have spent significant time in the field being better equipped to interpret these images.

## Conclusions

There is much to be gained by the successful employment of CT angiography in the evaluation of non-traumatic SAH. It is a quick and non-invasive procedure that can help guide clinical decision-making and provide an overall higher level of care for the patient. The perceived accuracy of this examination should be qualified based on the protocol details along with the experience and expertise of the radiologist reviewing the images. If a ruptured aneurysm is not detected on a CTA, dire consequences such as re-hemorrhage and death are possible. We propose that patients presenting with a non-traumatic, non-PMH SAH on head CT with a negative CTA should still be evaluated with catheter angiography in most circumstances.
